# Health-related behaviours and their relationship with self-rated health among Canadian adults

**DOI:** 10.1186/s12889-019-7249-4

**Published:** 2019-07-18

**Authors:** Natalie D. Riediger, Andrea E. Bombak, Adriana N. Mudryj

**Affiliations:** 1 Department of Food and Human Nutritional Sciences, Faculty of Agricultural and Food Sciences, University of Manitoba, 209 Human Ecology Building, Winnipeg, Manitoba R3T 2N2 Canada; 2 Department of Community Health Sciences, Max Rady College of Medicine, Rady Faculty of Health Sciences, University of Manitoba, Winnipeg, Manitoba Canada; 3 Department of Sociology, University of New Brunswick, Fredericton, New Brunswick Canada

**Keywords:** Self-rated health, Smoking, Fruit and vegetable, Sleep, Alcohol, Physical activity

## Abstract

**Background:**

Self-rated health (SRH) is a commonly used survey measure as a substitute for a clinical measure of health, which has demonstrated validity and reliability in a variety of populations. The referents that individuals incorporate into their self-evaluations have been shown to include health-related behaviours, though these relationships are not static. Our purpose was to describe and test for relationships between health-related behaviours and SRH among Canadian adults.

**Methods:**

We used pooled data from the Canadian Health Measures Surveys Cycles 3 (2012–13) and 4 (2014–15). All men and non-pregnant women aged 18 years and older were included (*n* = 6,789). We used binary logistic regression to test for relationships between health-related behaviours and SRH, including smoking status, adequate fruit and vegetable intake, inadequate sleep, alcohol use, and adequate physical activity.

**Results:**

The majority of respondents rated their health as good, very good, or excellent, though differences in SRH were found according to age group, highest level of household education, and income adequacy. Inadequate sleep was most strongly associated with poorer SRH among men and women combined, as compared to other health-related behaviours. Among women only, those who report heavy episodic drinking (OR, 2.64) or daily drinking (OR, 3.51) rated their health better, as compared to women who report low-risk alcohol use.

**Conclusions:**

Sleep quality is an important predictor of SRH for both men and women. Second, sex/gender differences must be considered in strategies to address alcohol use, as we may not be fully appreciating potentially health-affirming qualities associated with alcohol use among women.

## Background

Chronic diseases, including type 2 diabetes, cardiovascular disease, and cancer contribute significantly to mortality and morbidity in Canada [1]. Health-related behaviours such as fruit and vegetable intake, physical activity, smoking, alcohol use, and sleep, are significant predictors of many of these outcomes [2, 3]. The same health-related behaviours are also more common among low socioeconomic groups in Canada (Mudryj A, Bombak A and Riediger N: The relationships between health-related behaviours in the Canadian adult population, submitted to BMC Public Health-Under Review) and contribute to associated health disparities [1]. As such, these behaviours are often targets of health promotion policies and interventions. The majority of these population-level approaches have been educational campaigns/resources, as well as punitive measures such as taxation, age restrictions on purchasing, and restrictions on location of use. Neither approach has been particularly effective at addressing socioeconomic disparities in health-related behaviours. Health-related behaviours are not isolated phenomena but comprise routines and habits that make up a lifestyle [4], which are influenced by numerous broader social determinants of health and the context in which people live.

Furthermore, people do not necessarily incorporate ‘public health’ positions regarding health-related behaviours as inevitably positive or negative, and nor does everyone take up health messaging in the same way [5, 6]. The uptake of health messaging, in turn, may influence health-related behaviours in differing ways [7].

Self-rated health (SRH) is a commonly used measure in surveys as a substitute for a clinical measure of biomedical status or as a measure of health-related quality of life, which has demonstrated validity and reliability in a variety of populations [8, 9]. SRH is typically measured on a single-item asking respondents to choose a response that best describes their general health. The most common wording of the question is that included in the Medical Outcomes Study Short-Form-36 Health Survey (MOS SF-36) as follows: ‘In general, would you say your health is’ with the response items ‘excellent’, ‘very good’, good’, ‘fair’, or ‘poor’ [8–10]. Notably, there are limitations to a single-item measure as compared to a multicomponent measure as a global assessment of health, as a multicomponent measure has been shown to exhibit stronger relationships with life expectancy and chronic disease [11]. Despite the limitations, SRH has independently been shown to predict mortality [12–14], diabetes [12], and cardiovascular disease [15, 16]. Researchers have attempted to identify the referents that individuals incorporate into their self-evaluations that produce such consistent results. Results for health-related behaviours have varied and gender differences have been detected [17–19]. Importantly, unlike multicomponent measures, SRH reflects not only “objective” health, but is also a function of social norms and expectations regarding health. While for some research questions this may be problematic [11], the influence of social norms and expectations in how individuals refer to health-related behaviours in their SRH is an important component in understanding behavior, particularly in response to health messaging.

Health-related behaviours continue to be the subject of current public health priorities and policy development. For example, the Chief Public Health Officer’s Report on the State of Public Health in Canada 2018 has declared reducing alcohol use among Canadians as a public health priority [20]. Health Canada is also in the process of developing and rolling out their ‘Healthy Eating Strategy’ [21]. As social norms and expectations regarding health-related behaviours change over time, it is likely that referents that individuals incorporate into their SRH are also likely not static. In this way, a current exploration in the Canadian context is warranted. This could then give insight into the extent to which Canadians incorporate referents that are considered public health priorities. Therefore, we aim to test for relationships between health-related behaviours and SRH using a sex- and gender-based analysis.

## Methods

### Study design and sample

This study was completed using pooled data from the Canadian Health Measures Surveys Cycles 3 and 4. Cycle 3 was collected in 2012–2013 (*n* = 5,785) and Cycle 4 was collected in 2014–2015 (*n* = 5,794). Pooling was required for sufficient sample size and these are the two most recent surveys available for which pooling was possible due to existing weighting variables developed by Statistics Canada. Data collection methods, including survey sampling methods, for each cycle have been previously described [22]. Each sample was representative of the Canadian population in each time period, excluding individuals living in institutions, on-reserve, and full time members of the Canadian forces, and residents of certain remote regions.

We excluded participants < 18 years old and pregnant women (*n* = 2396 in Cycle 3 and *n* = 2394 in Cycle 4). All men and non-pregnant women aged 18 years and older were included, for a total *n* = 6,789. Data access for this study was approved by the Social Sciences and Humanities Research Council (SSHRC), which qualified for a waiver for approval from the University of Manitoba’s Health Research Ethics Board. All analysis were completed in keeping with Statistics Canada protocol.

### Measures

Socioeconomic variables included age group, sex, highest level of education, and income adequacy. *Age* was grouped as 18–29, 30–39, 40–49, 50–59, 60 years and older. *Highest level of household education* is grouped as: less than secondary school graduation, secondary school graduation, and post-secondary graduation. *Income adequacy*, as defined by Statistics Canada based on total household income and number of individuals in the household, is grouped as: lowest income group, lower middle income group, upper middle income group, and highest income group.

Health-related behaviours included smoking status, adequate fruit and vegetable intake, inadequate sleep, alcohol use, and adequate physical activity. Similar to previous research (Mudryj A, Bombak A and Riediger N: The relationships between health-related behaviours in the Canadian adult population, submitted to BMC Public Health-Under Review), responses have been mostly dichotomized. *Smoking status* is categorized as current smoker, former smoker, and never smoker. *Adequate fruit and vegetable* intake is defined as ≥4 times per day (not including potatoes or juice) and < 4 times per day [23]. *In*a*dequate sleep* is defined as 6 hours/day or less (‘short duration’), 10 h/day or more (‘long duration’), based on recent meta-analytic evidence [24], OR two or more of the following: having trouble going to sleep or staying asleep most or all of the time; never or rarely feeling refreshed by sleep; or find it difficult to stay awake during normal waking hours when you want most or all of the time [25]. *Alcohol use* is categorized as ‘heavy episodic drinking’, defined as ≥5 drinks for males or ≥ 4 drinks for females on one occasion ≥2 times per month over the past year [26], ‘daily alcohol use’, defined as consuming alcohol every day in the past year [26], or ‘low-risk alcohol use’. Respondents who reported both heavy episodic drinking and daily drinking, were categorized as ‘heavy episodic drinking’. *Adequate physical activity* is defined as the average of ≥30 min of low, moderate, or vigorous physical activity on 5 days per week [27]. *Number of positive health-related behaviours* were summed for each participant, totaling a value between 0 and 5. *Self-rated health* is categorized as excellent, very good, good, fair and poor. Specifically, participants were asked “how do you rate your health?”. SRH was further dichotomized as good health (excellent, very good, good) or poor health (fair, poor), similar to previous research [8, 28, 29].

### Analysis

Statistical analysis was completed using STATA. Significance was set at α = 0.05. Statistics Canada provided weights for the pooling of these two samples. Data were weighted using bootstrapping procedures for all data analyses [30]. This approximation technique is used to estimate standard errors, coefficients of variation, and confidence intervals for population-level estimates. A sex-stratified analysis was also completed.

First, SRH was described according to age group, education, income adequacy, and each health-related behavior using descriptive statistics, and stratified by sex. Differences in SRH according to the previously listed variables were tested using Chi-square test. Second, binary logistic regression was used to test for a relationship between number of positive health-related behaviours and SRH, adjusting for age group, sex, income adequacy, and highest-level of household education. Third, binary logistic regression was also used to test for relationships between each health-related behavior and SRH, adjusting for age group, income, and education. Lastly, we included a model with all health-related behaviours to test for their independent associations with SRH, given that we have previously reported that some health-related behaviours are associated with each other (Mudryj A, Bombak A and Riediger N: The clustering of health-related behaviours in the Canadian adult population, submitted to BMC Public Health-Under Review).

## Results

Overall, Canadians rated their health well and there was not a significant gender difference; 88.4% (SE, 0.8) of men and 88.6% (SE, 1.0) of women rated their health as good, very good, or excellent. Income adequacy and household education were positively and significantly associated with better SRH (Table 1). Age group was negatively associated with better SRH. Each positive health-related behavior was significantly associated with better SRH for both men and women using Chi-square tests, with the exception of alcohol use. For men, SRH was not significantly different according to alcohol use. For women who reported heavy episodic drinking, 96.9% rated their health as good, very good, or excellent, and 94.2% of women who reported daily drinking rated their health similarly; in contrast, 87.3% of women who reported low-risk alcohol use reported good, very good, or excellent health.

**Table 1:** Self-rated health (good, very good, excellent) by demographic and socioeconomic characteristics, and health-related behaviours

	Men and Women	*p*-value^a^	Men only	*p*-value	Women only	*p*-value
Age (y)						
18–29	90.7 (0.7)	< 0.001	94.0 (1.3)	< 0.001	87.4 (2.7)	< 0.05
30–39	93.1 (1.0)		89.8 (2.6)		96.7 (1.3)	
40–49	90.1 (0.6)		92.8 (1.1)		87.5 (2.8)	
50–59	84.8 (0.8)		82.9 (2.5)		86.7 (2.8)	
60+	84.6 (0.7)		82.9 (2.0)		86.1 (1.6)	
Income adequacy						
Lowest income	79.1 (3.1)	< 0.001	81.1 (4.8)	< 0.01	77.1 (4.3)	< 0.001
Lower-middle income	82.7 (1.6)		83.3 (3.0)		82.3 (2.1)	
Upper-middle income	86.2 (1.4)		83.0 (1.9)		87.3 (2.4)	
Highest income	92.4 (0.9)		92.0 (1.0)		92.7 (1.4)	
Household Education						
< Secondary School	73.9 (3.6)	< 0.001	77.5 (4.7)	< 0.01	70.2 (5.9)	< 0.001
Secondary School Graduation	85.4 (1.7)		87.1 (2.3)		83.4 (2.6)	
Post-Secondary School Graduation	90.2 (0.6)		90.7 (1.0)		90.7 (1.0)	
Smoking status						
Current smoker	83.1 (0.7)	< 0.01	82.9 (2.3)	< 0.01	83.3 (2.7)	< 0.01
Former smoker	86.7 (1.4)		86.1 (1.7)		87.5 (2.0)	
Never smoker	91.8 (0.8)		93.1 (1.0)		90.8 (1.1)	
Adequate fruit and vegetable intake						
Yes	90.9 (1.0)	< 0.01	91.1 (1.5)	< 0.01	90.8 (1.1)	< 0.05
No	87.4 (0.6)		87.5 (0.8)		87.3 (1.3)	
Adequate sleep						
Yes	92.2 (0.7)	< 0.001	92.0 (1.0)	< 0.001	92.5 (1.)	< 0.001
No	81.8 (1.2)		81.8 (1.6)		81.8 (1.8)	
Alcohol use						
Heavy episodic drinking	92.1 (2.5)	< 0.05	89.0 (4.2)	0.524	96.9 (1.5)	< 0.01
Daily drinking^b^	91.9 (1.3)		90.6 (1.6)		94.2 (2.3)	
Low-risk alcohol use	87.6 (0.7)		88.2 (0.8)		87.3 (0.7)	
Adequate physical activity						
Yes	89.6 (0.6)	< 0.001	88.3 (1.0)	< 0.001	90.2 (0.9)	< 0.001
No	83.4 (1.6)		83.7 (1.4)		82.0 (1.0)	

^a^Chi-square test

^b^Excluding daily drinkers who also report heavy episodic drinking

The number of positive or protective health-related behaviours reported was not associated with SRH using binary logistic regression (Table 2). The lack of significant relationship existed in unadjusted analysis and persisted in both sex-stratified analysis and adjusted analysis.

**Table 2:** Odds ratio (95% confidence interval) of good, very good, or excellent self-rated health according to number of positive health-related behaviours

Number of positive behaviours	Model 1	Model 2	Model 3	Model 4	Model 5
0	Reference	Reference	Reference	Reference	Reference
1	0.42 (0.15–1.19)	0.49 (0.19–1.31)	0.42 (0.40–1.22)	0.49 (0.13–1.89)	0.52 (0.07–3.50)
2	0.70 (0.23–2.11)	0.87 (0.31–2.44)	0.75 (0.22–2.47)	1.28 (0.32–5.15)	0.58 (0.09–3.42)
3	1.00 (0.37–2.76)	1.27 (0.50–3.23)	1.03 (0.34–3.10)	1.36 (0.35–5.32)	0.97 (0.15–5.90)
≥ 4	1.78 (0.60–5.24)	2.23 (0.82–6.05)	1.61 (0.50–5.23)	2.22 (0.69–14.96)	1.15 (0.18–7.44)

Model 1: Unadjusted

Model 2: Adjusted for age group and sex

Model 3: Adjusted for age group, sex, income adequacy, and highest level of education

Model 4: Men only, adjusted for age group, income adequacy, and highest level of education

Model 5: Women only, adjusted for age group, income adequacy, and highest level of education

Smoking, inadequate fruit and vegetable intake, inadequate sleep, and inadequate physical activity were each adversely and significantly associated with SRH, independent of sex, age group, income adequacy, and household education (models 1–5, Table 3). When including all health-related behaviours in the same model (model 6), odd ratios changed little. Notably, fruit and vegetable intake was no longer significant after adjustment.

**Table 3:** Odds ratio (95% confidence interval) of good, very good, or excellent self-rated health according to adverse health-related behaviours

Health-related behaviour	Model 1–5, adjusted^a^	Model 6, adjusted^b^	Model 7, men only, adjusted^c^	Model 8 women only, adjusted^c^
Smoking				
Never	Reference	Reference	Reference	Reference
Former	0.71 (0.48–1.04)	0.68 (0.46–1.01)	0.66 (0.37–1.16)	0.68 (0.38–1.20)
Current	0.53 (0.35–0.79)^e^	0.56 (0.38–0.81)^e^	0.44 (0.25–0.77)^e^	0.67 (0.38–1.18)
Inadequate fruit and vegetable intake	0.75 (0.57–0.99)^d^	0.80 (0.60–1.09)	0.86 (0.50–1.48)	0.77 (0.54–1.10)
Inadequate Sleep	0.40 (0.29–0.53)^f^	0.41 (0.30–0.57)^f^	0.44 (0.28–0.69)^e^	0.41 (0.28–0.60)^f^
Alcohol use				
Low-risk	Reference	Reference	Reference	Reference
Heavy episodic drinking	1.37 (0.97–1.90)	1.65 (1.13–2.42)^d^	1.22 (0.76–1.98)	2.64 (1.09–4.51)^d^
Daily drinking	1.86 (0.91–3.80)	1.82 (0.86–3.84)	1.39 (0.55–3.51)	3.51 (1.68–6.37)^e^
Inadequate Physical Activity	0.57 (0.42–0.77)^f^	0.64 (0.47–0.87)^e^	0.68 (0.43–1.60)	0.58 (0.37–0.93)^d^

^a^Presented as five separate logistic regression models for each health-related behaviours, adjusted for age group, sex, income adequacy, and highest level of education

^b^Presented as one logistic regression model including all health-related behaviours, adjusted for age group, sex, income adequacy, and highest level of education

^c^Presented as one logistic regression model including all health-related behaviours, adjusted for age group, income adequacy, and highest level of education

^d^< 0.05, ^e^ < 0.01, ^f^ < 0.001

Interestingly, alcohol use was not associated with SRH in unadjusted, sex-pooled analysis. However, sex-stratified analysis indicated no relationship between heavy episodic drinking or daily drinking and SRH among men (model 7, Table 3), but an odds ratio of 2.64 for heavy episodic drinking and 3.51 for daily drinking in reporting good, very good, or excellent SRH among women (model 8, Table 3). Other than alcohol use among women, inadequate sleep and physical activity persisted in its significant relationship with SRH in sex-stratified analysis.

## Discussion

In the present study, participants who reported positive health-related behaviors were significantly more likely to rate their health as good, very good, or excellent in unadjusted analysis, with the exception of alcohol use. Income adequacy and highest-level of household education were also significantly and positively associated with better SRH. Though SRH was not significantly different according to sex in the present study, there were sex-specific relationships between health-related behaviours and SRH, despite previous research indicating that women and men interpret and assign a similar meaning to SRH [31]. Sex differences were most notable for alcohol use and SRH, such that women who reported heavy episodic drinking had 2.64 times higher odds of reporting good, very good, or excellent SRH, independent of age group, income adequacy, highest level of household education, and other reported health-related behaviours. Similarly, women who report daily drinking demonstrated a 3.51 times higher odds of better SRH, adjusting for the same variables.

The positive relationship between socioeconomic status and SRH in the present study is consistent with the literature in this area across different populations [32, 33]. However, the significant relationship between several health-related behaviours (inadequate sleep, inadequate physical activity, and smoking status) and worse SRH persisted when accounting for income adequacy and highest level of household education. Notably, the relationship between current smoking and SRH was attenuated when further adjusting for education and income, as well as other health-related behaviours. This finding is aligned with our previous research using the same data, such that current smoking status exhibits a significant socioeconomic gradient and is associated with all other health-related behaviours among men (Mudryj A, Bombak A and Riediger N: The clustering of health-related behaviours in the Canadian adult population, submitted to BMC Public Health-Under Review). SRH is partially based upon the health information available to an individual [34]. Previous research also suggests that participants with higher levels of education are more likely to refer to health behaviors in their assessment of SRH [35]. Unfortunately, we were not able to explore a mediating role for socioeconomic status in the present study given the sample size limitations.

Inadequate fruit and vegetable intake was independently associated with SRH in sex pooled analysis. Our results suggest that fruit and vegetable intake is generally incorporated into Canadian’s referents for health. Canadians frequently mentioned fruits and vegetables when discussing what constituted a healthy diet [36], which likely indicates that the educational campaigns that extolled the health benefits of fruit and vegetable intake in Canada to which they were exposed were received. However, this has not translated into higher fruit and vegetable intake at the population level (Mudryj A, Bombak A and Riediger N: The clustering of health-related behaviours in the Canadian adult population, submitted to BMC Public Health-Under Review). Further educational efforts, such as the new Canada’s Food Guide, should be viewed skeptically. Individuals may not be ignorant of institutionalized dietary recommendations, as research has shown that Canadians have a high level of awareness of the Food Guide [37]. Rather their diets may reflect material resources, pleasure, and enjoyment in precarious and disadvantaged circumstances.

Alcohol use has demonstrated divergent patterns with SRH in various populations. In a sample of community-based older English adults, no pattern of alcohol consumption was associated with SRH; also any association between alcohol use and SRH was not influenced by socioeconomic status [38]. Among Spanish older adults moderate drinking was associated with better SRH; heavy drinking was not associated with SRH [39]. In a sample from Estonia, alcohol abstainers were more likely to report poor SRH as compared to people drinking somewhat frequently [40]. It is possible that alcohol abstainers are more likely to have pre-existing health issues, as reported by Abuladze and colleagues [40], which may introduce bias into the comparison between high-risk alcohol use and SRH in the present study. Indeed, a Canadian study by Segovia and colleagues [41] reported an inverted U-shaped relationship between alcohol intake and better SRH; thus, grouping alcohol use into fewer categories potentially groups together heterogeneous drinking patterns. The same authors also reported that binge-drinking appeared to worsen perceived health status, though sex differences were not explored [41]. This finding from a Newfoundland sample, approximately 30 years ago, compared to the present study may indicate changes in perceptions of the health effects of binge-drinking over time in Canada, particularly among women. A 2005 Canadian study among older adults, also demonstrated, like the present study, that frequent, moderate alcohol intake was associated with better SRH compared to non-drinkers and infrequent drinkers, among both men and women in sex-stratified analysis [42].

Sex-stratified analysis in the present study further indicated that the positive relationship between heavy episodic drinking and better SRH was limited to women. This is in contrast to the finding previously reported by Mariconi and Nadeau among Canadian older adults, where the associations between drinking patterns and SRH, collected in 2005, were similar between men and women [42]. The main differences between the former study and the present study are the age groups included and also the time period, which indicates that perhaps younger age groups may be exhibiting different sex-based referents for alcohol and SRH.

SRH is an inherently holistic measure and previous qualitative research conducted in Canada indicated that alcohol use is associated with many social activities [43]. Therefore, it is possible that women drink more for social reasons and perceive health benefits from the associated social networks. Indeed, research reviewed by Wilsnack and colleagues [44] indicates differences in reasons men and women drink. Notably, research from New Zealand has indicated substantially different drinking patterns between men and women related to location of purchase, beverage choice, and time of consumption [45], which may also reflect differences in social settings. Furthermore, men are consistently found to report more problem drinking across many countries [44] and problem drinking often co-occurs with mental health issues among men [46]. Together this may explain the lack of relationship between heavy episodic drinking or daily drinking, and SRH among men in the present study.

While the positive relationship between heavy episodic drinking and better SRH may be due to positive aspects associated with drinking, it may also be due to limited concern about “high-risk” alcohol use. In an Ottawa study, only 56% of respondents were concerned or strongly concerned about binge drinking; it was selected least frequently among alcohol-related concerns to be addressed first. Of those who cited binge-drinking as a concern, this concern was attributed to worries centered on individual risk, vulnerability to violence, and risk of future addiction [43].

Adequate sleep was the only health-related behavior that was significantly associated with SRH for both men and women in sex-stratified analysis in the present study, and similarly so. This can be partially attributed to the limited associations between inadequate sleep to the other health-related behaviours such that other health-related behaviours were no longer significantly associated with SRH after their adjustment. However, this finding also likely speaks to the importance of mental health and SRH given the consistent associations between sleep quality and mental disorders [47], and also the trajectory of mental health symptoms [48]. Others have also reported significant relationships between both short- and long- sleep duration and poor SRH, including in Canada [41]. Notably, we have previously reported that 35.5% of men and 36.5% of women report inadequate sleep (Mudryj A, Bombak A and Riediger N: The relationships between health-related behaviours in the Canadian adult population, submitted to BMC Public Health-Under Review). We are not aware of any public health policies and health promotion programs focused on addressing sleep quality. Given the increasing focus on patient-oriented research in Canada [49], it may be worthwhile to further examine if/how health promotion policies may improve the health behaviour most strongly associated with SRH among Canadians, namely, sleep, and related mental health issues.

The study is subject to several limitations. First, the small sample size limited our ability to disaggregate further and analyze age- or socioeconomic-specific relationships. Second, the survey is also subject to non-response bias; specifically, the response rate for both surveys was 74 and 76%, respectively [30]. Third, all health-related behaviours are self-reported and thus are subject to under- or over-reporting. Lastly, by dichotomizing and categorizing health-related behaviours much information has been lost and relationships with SRH may be different based on how health-related behavior variables have been grouped.

## Conclusions

In conclusion, public health priorities may not align with Canadians’ referents for SRH, a known powerful predictor of morbidity and mortality. Firstly, the role of sleep in health and wellbeing is likely underestimated, or at the very least, under-actioned. Further research is needed to examine how public health interventions may improve sleep quality, particularly related to mental health, as the literature suggests is related to sleep quality. Second, gender differences must be considered in future research related to alcohol use. Women do not appear to be utilizing health messages related to alcohol in their health referents. It is possible we may not be fully appreciating potentially health-affirming qualities associated with alcohol use or coping aspects of alcohol use among Canadian women. Further qualitative research is required to explore alcohol use among women, particularly in light of recent reports of increased rates of alcohol-related hospitalizations for Canadian women [50], and the recent recommendation, published in Lancet, that no amount of alcohol is safe [51]. The relationships between alcohol use and overall health and well-being is likely nuanced, and strategies aimed at addressing alcohol use among woman must take these nuances into consideration.

## Data Availability

The datasets used and analyzed during the current study are available by permission from Statistics Canada and the Canadian Research Data Centre Network. Please contact the Canadian Research Data Centre Network or your local Research Data Centre for more information (https://crdcn.org/find-a-rdc).
